# Granite outcrops as possible havens for biodiversity in arid land

**DOI:** 10.3897/BDJ.12.e137043

**Published:** 2024-11-15

**Authors:** Mohammed Shakdofa, Omar Almaghrabi, Emad A Alsherif

**Affiliations:** 1 University of Jeddah, Jeddah, Saudi Arabia University of Jeddah Jeddah Saudi Arabia; 2 Beni-Suef University, Beni Suef, Egypt Beni-Suef University Beni Suef Egypt

**Keywords:** diversity, arid land, inselbergs, vegetation, Saudi Arabia, flora

## Abstract

Little is known about granite outcrops in arid regions. The current study sought to determine if granite outcrops generate wetter islands compared to their arid surroundings. Forty independent phytosociological stands dealing with granite outcrops (25 stands) and nearby matrix (15 stands) vegetation were sampled. A total of 146 species of vascular plants were identified, categorised into 39 families and 106 genera. Results recorded more taxa in granite outcrops than in their matrix; specifically, there were 29.2% more species, 33.8% more genera and 26.6% more families in the granite outcrops than in their matrix. Only 16 species were reported in the matrix area that are not present in the granite outcrops, compared to 46 species found in the granite outcrops that are absent from the matrix area. The ratios of therophytes and phanerophytes in granite outcrops were 5% and 11.1% greater, respectively, than those observed in their matrices. The proportions of Sudano-Zambasian elements were higher in granite outcrops than those in the matrix and, in contrast, the Sudano-Arabian and tropical elements proportions were higher in the matrix than those in the granite outcrops. The current results concluded that granite outcrops form wetter island-like habitats as compared to their surroundings.

## Introduction

Granite outcrops take the form of low mountains or dome-shaped hills and disc-shaped pavements with a wide range of temperatures and biomes (Porembski and Barthlott 2000, Twidale and Vidal Romaní 2005). The erosive processes in rock outcrops are slower than in the surrounding area, making these landforms remnant characteristics that are frequently quite old (Bremer and Sander 2000). Granite outcrops are called inselbergs because inselbergs act as island-like ecosystems due to distinctive ecological conditions from the surroundings (Ornduff 1987). Typically, they have clearly defined borders where plant assemblages and ecological conditions vary significantly from their surroundings (Porembski and Barthlott 2000, McGann 2002). According to Porembski and Barthlott (2000), granite inselbergs' environmental conditions are generally harsh on plant life and can vary significantly over short distances, creating variability that promotes plant diversity (Schut et al. 2014).

According to Kottek et al. (2006), Saudi Arabia is classified as a desert zone. Its topography is made up of many physiographic zones, including lava fields (Harrats), salt flats (Sabkhahs), valleys, slopes and mountains with sharp or flat summits. Except for Asir Province, most of Saudi Arabia's climate is classified as having "less rainy climates" according to Koppen and as an "arid province" according to Thornthwaite (Al-Nafie 2008). Several studies have demonstrated that the region's topography and climate are the primary factors influencing the degree of speciation (El-kady et al. 1995, Shaltout and Mady 1996, Shaltout et al. 1997, Alsherif et al. 2013). Saudi Arabia includes plenty of rocky outcrops and ecologists may easily observe that the flora on these sites is distinct from that of the surrounding region (Alsherif and Fadl 2016, Fadl et al. 2021). It was documented that identifying plant variety patterns on granite inselbergs and their biogeographical, environmental and spatial correlates is a crucial issue for conservation biogeography globally, given the growing concerns about mining, grazing, urbanisation, water harvesting and invasion of weeds (Porembski et al. 2016). Many previous studies reported that granite outcrops are typically studied in wet environments, where they exist as dry islands within a humid vegetation matrix (Bremer and Sander 2000, Porembski and Barthlott 2000, Twidale and Vidal Romaní 2005). In contrast, less is known about rock outcrops in arid regions (de Paula et al. 2020); hence, the current study sought to answer the question of whether granite outcrops form wetter island-like habitats as compared to their surroundings.

## Material and methods

**Figure 1.**
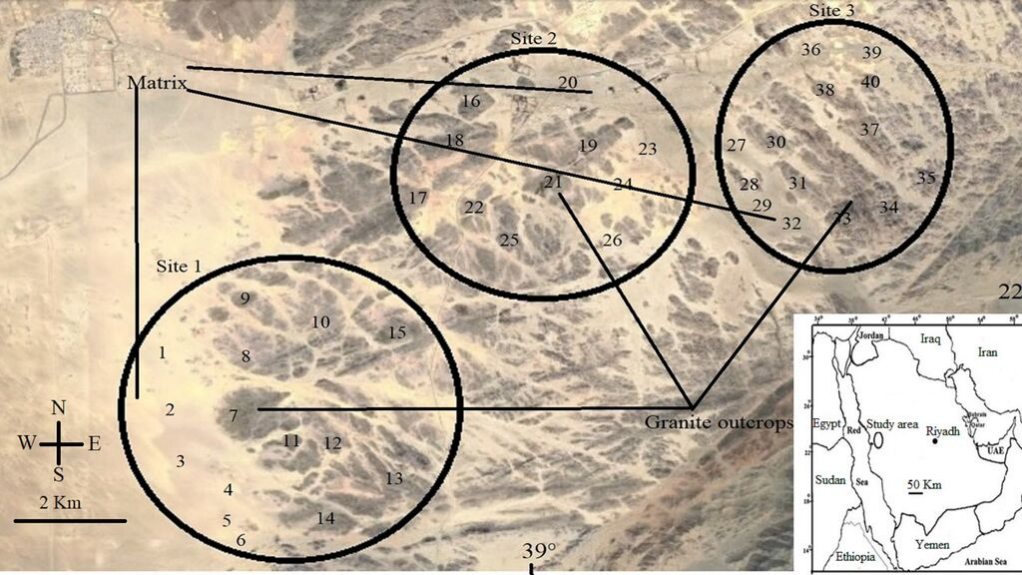
Map showing location of the study area and sampling stands.

**Figure 2.**
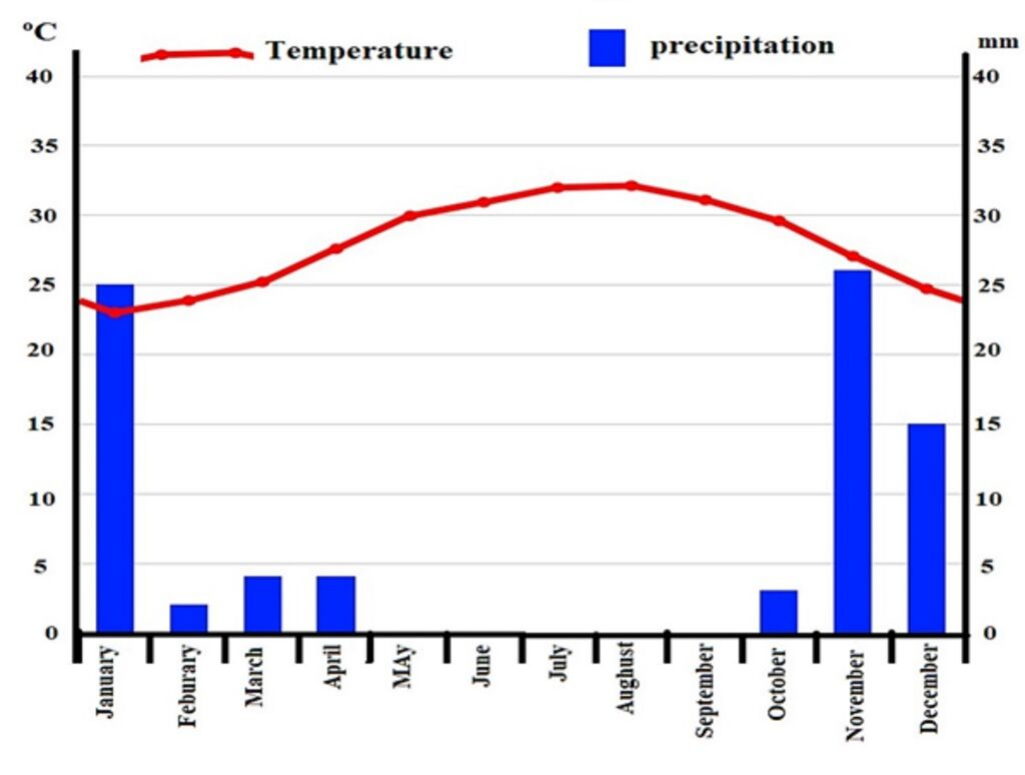
Averages temperature (ºC) and precipitation (mm) of the studied area.

The study area is located between longitudes 39°20' and 39°27' to the east and latitudes 22°5' and 22°18' north within the territory of the Arabian Shield (Fig. 1). The climate is characterised by high temperatures in summer and warm in the winter (Fig. 2). According to the Köppen climatic classification, it is a warm desert climate (type BWh), with hot, dry summers and moderate, mostly dry winters. The rainfall, apart from its scarcity, is irregular and variable, ranging between 0 to 70 mm. In the last two years, heavy but sporadic rainfall occurred on one or two days. The study area has numerous granite rock outcrops, including Pan-African calcalkalic and alkalic granites, which vary in area and height and stand out from the surrounding area due to various plant species (Alsherif et al. 2013). Sampling of the floristic composition was focused on granite and their nearby matrices, in which matrix habitats were different wadies with open vegetation and sandy soil. Three sampling sites were selected; these sites are only 10 km apart and, therefore, experience the same climatic conditions. At each site, there are a large number of isolated granite patches that form areas of variable dimensions (0.6–1.5 km long × 0.2–1.3 km wide), isolated within a large area of sandy soil and these were selected. To represent the ecological heterogeneity found in both sampling localities, a total of 40 independent phytosociological releves dealing with granite outcrops (25 stands) and nearby matrix (15) vegetation were sampled. The vegetation sampling was done in accordance with the Zürich-Montpellier School of Phytosociology, avoiding areas with a high degree of disturbance (i.e. overgrazing). Due to the challenges of studying the phytosociology of chasmophytic vegetation, the Ortiz and Rodríguez-Oubiña (1993) methodological proposal was adopted in this survey, which involved treating each outcrop as a stand made up of a variety of biotopes (i.e. fissures, crevices and cavities). The selected stands varied in size and shape since the outcrop limits were established using geomorphologic and topographic parameters. An independent plot measuring 5 m x 5 m was created in each outcrop to account for the local heterogeneity of each outcrop. All vascular plants in each 25 m^2^ plot were counted. Data were gathered from January 2019 to December 2022 during the growth season. The plant specimens were identified and named according to Collenette (1999) and Chaudhary (2001). The life forms of the collected species were determined by the location of the regenerative buds and the portions lost during the unfavourable season (Braun-Blanquet 1932). The biogeographic affinities of species were determined according to Zohary (1973).

## Data resources

List of species recorded in the different studied location is available here https://figshare.com/s/7e41acc625ff5e0d21d7.

## Results

### Characteristics of granite outcrops and their matrix flora

**Figure 3.**
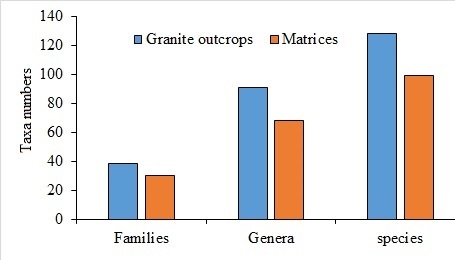
Taxa numbers in granite outcrops and their matrices.

During the fieldwork, we recorded a total of 146 vascular plant taxa distributed amongst 39 families and 106 genera (Table 1). The most common families found were Poaceae (16 species), Fabaceae (14) and Zygophyllaceae (11) which together accounted for 28% of all the species (Table 1). Other common families recorded were Euphorbiaceae (10 species), Asclepediacea and Capparaceae (seven species each). *Euphorbia* was the most common genus with seven (Fig. 3) species followed by *Tribulus* with five Fig. 3) species. Taxa numbers in granite outcrops were more than those in their matrix (Fig. 3), where species numbers in granite outcrops exceeded those of matrix by 29.2%, genera by 33.8% and families by 26.6%

Forty-six species were recorded in the granite outcrops that are not present in the matrix area, while only sixteen species were recorded in the matrix area that are not present in the granite outcrops. *Commiphoragileadensis*, *C.quadricincta*, *Euphorbiacuneata*, *Lavandulacoronopifolia*, *Grewiaerythraea* and *Carallumaacutangular* were the most frequent species in granite outcrops and absent in the matrix. In contrast, *Rhazyastricta*, *Prosopis Juliflora*, *Daturainnoxia*, *Acaciaehrenbergiana* and *Acaciatortilis* were the more frequent species in the matrix and absent from the granite outcrops (Table 1).

### Vegetation of the granite outcrops and their matrix

**Figure 4.**
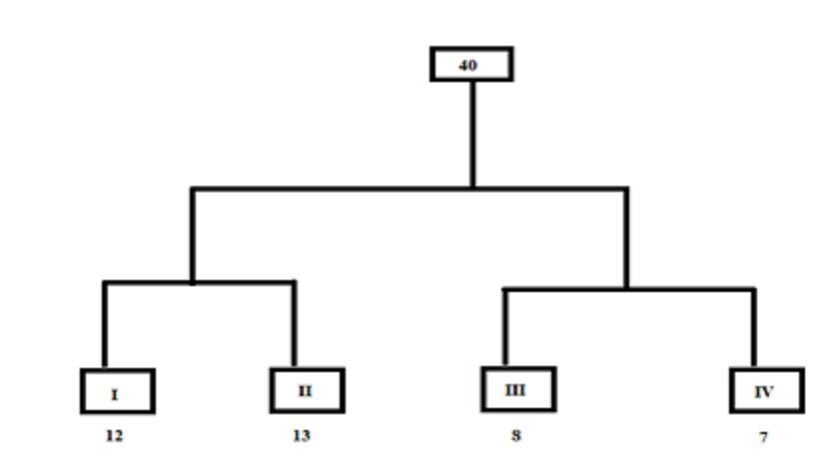
Twinspan analysis, resulting in the simplification of 40 stands into four vegetation groups. Granite outcrop vegetation is represented by groups I and II, whilst matrix vegetation is represented by groups III and VI.The number of stands in each group is shown beneath each group code.

When it is dry, the outcrops' vegetation is at its least noticeable and the stony, boulder-strewn slopes seem unduly bleak to the untrained eye. However, upon closer examination, little shrubby trees that are devoid of any leaves can be found widely spaced across the outcrops. Two main groups were obtained from the WARD classification (Fig. 4). The first group included the stands from the granite outcrops, while the second group included the stands from the matrices. Each group is further divided into two subgroups to form four vegetation groups as follows:

- **Vegetation of group I**

Group I is characterised by several *Acacia* spp., *Euphorbiacuneata* and *Commiphoragileadensis* trees in the granite outcrops. On the lower slopes, the evergreen shrub *Cadabaglandulosa* stands out with its dark green leaf. This bush is often paired with *Lyciumshawii* and *Carallumaacutangula*. The most prevalent tree on the lower hills was *Acaciahamuslosa*, followed by *Euphorbiacuneata* and *Commiphoragileadensis*. An abundance of *Pennisetumdivisum*, *Stipacapensis* and *Blepharisattenuate*, gave the slopes a hint of green (Fig. 4).

- **Vegetation of group II**

Vegetation of this group was dominated by *Cymbopogonschoenanthus* community. The woody herbs which created after rainfall an incredibly beautiful natural rockery, include the compact colonies of *Cleomebrachycarpa*, *Cleomeamblyocarpa* and *Aervajavanica*, all of which have white woolly flowers.

- **Vegetation of group III**

Leguminosae was well represented with *Indigoferaspinosa* in this group, the most characteristic perennial herb of the hills and rubble plains being *I.coerulea*, *Tephrosianubica*, *T.encomptosperma* and *Sennaholosericea*. The large family Compositae, which is so dominant in central Arabia, is present, but poorly represented by *Dicomaschimperi*. Zygophyllaceae, by contrast, is one of the most important groups with *Zygophyllumsimplex*, *Tribuluslongipetalus* and *Fagoniaparviflora* all being common. The Labiates include *Leucasinflata*, *Salviaaegyptiaca* and *Lindenbergiasinaica*. Other common plants which were identified include *Corchorusdepressus*, *Polygalairregularis*, *Morettiaparvifiora* and *Aervajavanica*. Large trees may form a continuous fringe around larger watercourses, with growth occurring closer to drainage lines. *Maeruacrassifolia* small trees are seldom seen, whereas *Lyciumshawii* plants are frequently found. There are other species of *Acacia*; *A.ehrenbergiana* and *A.raddiana*, for instance, are primarily found in the shallow drainage lines. The edges of the large watercourses are home to all these species, as well as large trees of several unidentified *Acacia* species.

- **Vegetation of group IV**

The most prevalent species is *Cleomepallida* followed by *Lasiurus* and *Stipacapensis*. Although it is present here, *Panicumturgidum* is not as widely dispersed as it is in other places. *Commicarpusboissieri*, *Corchorusdepressus*, *Euphorbiagranulata*, *Morettiaparvifiora*, *Fagoniaparvifiora*, *Tribuluslongipetalus* and *Indigoferaspinosa* are common species. On large sandy mounds, however, there was a much-interrupted fringe made up of *Leptadeniapyrotechnica*, *Calotropisprocera* and the usual *Acaciatotilis*, *ZiziphusspinaChristi* and *Salvadora*. In addition, beds of grass called *Pennisetumsetaceum*, *Rhazyastricta* and *Aervajavanica* were recorded. In contrast to the coastal plain, the *Cleomepallida Panicumturgidum* connection was more confined and poorly developed in the deep, dusty alluvium that floodwaters deposit on the plain.

The evergreen shrub *Cadabaglandulosa* was particularly abundant in this group and it was found at least sparingly in flower at any time of the year. *Abutilonpannosum* and *A.fruticosum* were characteristic of this habitat and also was found with green leaves and even in flowers far into the dry season. Tussocks of *Panicumturgidum* and cushions *of Cymbopogonschoenanthus* were recorded in the silty gullies. Other typical plants of this habitat were *Farsetialongisiliqua*, *Colocynthisvulgaris*, *Pergulariatomentosa* and *Ochradenusbaccatus*. Shrubby trees of both *Acaciaehrenbergiana* and *A.tortilis* were also frequent in the lower reaches of the watercourses.

### Life forms and their distribution

**Figure 5.**
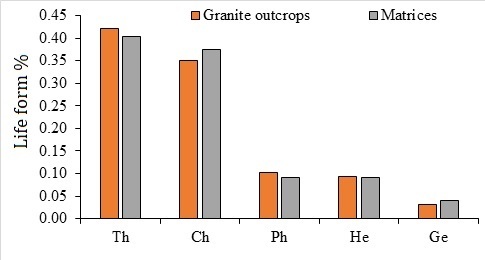
Life forms (%) in granite outcrops and their matrices. Ph, phanerophytes; Ch, chamaephytes; G, geophytes; He, hemi-cryptophytes and Th, therophytes.

Five life forms were recorded in the study area: therophytes, chamerophytes, phanerophytes, hemicryptophytes and geophytes. The therophytes dominated the others by 39.7 %, followed by chamerophytes which exhibited 36.9% of the recorded life forms (Fig. 5). Phanerophytes recorded 11.6%, while geophytes recorded the lowest ratio with 2.7%. The results show the distribution of the different life forms in granite outcrops and their matrix, indicating that the proportions of therophytes and phanerophytes in the granite outcrops were 5% and 11.1% higher, respectively, than those reported in their matrices (Fig. 5). In contrast, the proportions of chamaephytes and geophytes exceeded those in the matrix by 5.7% and 33.3%, respectively, while hemicryptophytes recorded the same percentages in both granite outcrops and their matrices.

### Chorological affinities and their distribution

**Figure 6.**
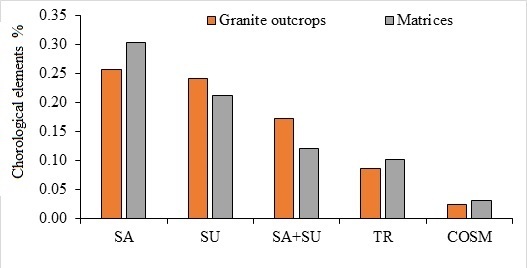
Percentages of the main chorological elements in granite outcrops and their matrices. SA, Saharo-Arabian; SZ, Sudano-Zambezian; TR, Tropical and COSM, cosmopolitan.

Fig. 6 shows that the monoregional species, Saharo-Arabian, dominate the studied area by 76.7%, where Saharo-Arabian elements alone represented 39.7% of the total recorded species, followed by the Sudano-Zambesian elements (by 36.9%). Approximately 11.6% of the sampled species were biregional, with their distributions extending across the Saharo-Arabian and Sudano-Zambesian regions. The obtained data show that the proportion of the Saharo-Arabian, tropical and cosmopolitan elements in the matrix habitats are more than those in granite outcrops by 25%, 12.5% and 50%, respectively (Fig. 6). In contrast, the Sudano-Zambesian and the bioregional Saharo-Arabian and Sudano-Zambesian elements decreased in the surrounding habitat by 14.2% and 41.6%, respectively.

## Discussion

The current results showed that granite outcrops have a different and richer floristic composition than the surrounding matrix vegetation. Although the current study's granite outcrops are quite modest in comparison to a prior survey that covered an area ten times larger (Alsherif et al. 2013), the number of species documented in the current study accounts for 18.1% of the previous study's total. Additionally, 31.5% of the species recorded in the granite outcrops were absent from the surrounding habitat. The study area is exposed to long periods of severe drought and scarcity of rainfall, while the granite rocks in the area cause water vapour to condense, which increases the water content of the soil between the cracks of the granite rocks and provides the opportunity for the growth of some plants that cannot grow in the adjacent area due to its extreme drought (Hopper et al. 1997). Typically, granite outcrops have distinct borders with plant communities and ecological conditions that differ greatly from their surrounds (Porembski and Barthlott 2000, McGann 2002). These findings highlight the significance of the granite outcrops in arid lands as distinct environments, frequently characterised by less stressful (wetter) conditions than the surrounding matrix. It was documented that climate stability and time-integrated areas, on the other hand, serve as the foundations for both terrestrial and marine global biodiversity patterns (Fine 2015).

The obtained results agree with many previous studies in wet environments. Carvallo et al. (2019) reported that granite outcrops are characteristic formations that contrast with the surrounding matrix and may support a unique flora. In addition, Nie et al. (2018) and Corlett and Tomlinson (2020) reported that granite outcrop habitats can be thought of as climatic, hydrological and edaphic islands. Previous studies found a great diversity of species in granite outcrop flora (Didukh and Vasheniak 2018,Carvallo et al. 2019, Kontopanou and Panitsa 2020). Previous studies have stated that granite outcrops can provide defence against disturbances like fire and grazing (Gomes et al. 2014, Nie et al. 2018). In our study, we consider that granite outcrops are considered a defence from severe drought.

It is possible that, due to their ability to live in arid climates, the therophytes' dominated the recorded life forms, followed by chamaephytes, in this study's life-form analysis. Therophytes amongst the ephemerals are greater drought-escapers than other life forms. As therophytes restrict how much water they take in and release during the hot summer months, their proportion was higher in granite outcrops than in the surrounding matrix. In contrast, the proportion of chamaephytes in the surrounding matrix was higher than its proportion in the granite-outcrops, which could be attributed to their being more resistant to summer dryness than therophytes (Lee et al. 2021). Regarding the recorded chorological elements, the Sudano-Zambesian and Saharo-Arabian components have the greatest chorological elements. These chorotype elements are well adapted to harsh habitats like intense heat and aridity. The percentage of Sudano-Zambezian elements in granite outcrops was higher than its percentage in the surrounding matrix and, in contrast, the percentage of the Saharo-Arabian elements in the matrix was higher than its percentage in granite outcrops. The previous observations can be explained due to that Saharo-Arabian elements which are more tolerant to drought than Sudano-Zambezian elements (Alsherif et al. 2013).

## Conclusions

Our study is the first attempt to describe the differences between the vegetation of granite outcrops and their nearby matrix in arid lands. The present results highlight the importance and uniqueness of granite outcrop environments that contain higher taxa. Future research should also take into account the importance of outcrop-scale environmental gradients in arid lands and highlight their importance to plant diversity.

## Figures and Tables

**Table 1.** Species list recorded in the granite outcrops and their matrices with their families, life forms and chorology. The life forms are Ph: phanerophytes, Ch: chamaephytes, G: geophytes, He: hemicryptophytes and Th: therophytes. The chorotypes are: COSM: cosmopolitan, AM: American, IT: Irano-Turanian, ME: Mediterranean, SA: Saharo-Arabian, SU: Sudano-Zambezian and TR: Tropical. 1: present, 0: absent.

**Species**	**Families**	**Habitat**		**Life form**	**Chorology**
		**Granite outcrop**	**Matrix**		
*Blepharisattenuata* Napper	Acanthaceae	1	1	Ch	IT+SA
*Abutilonfruticosum* Guill. & Perr.	Malvaceae	1	1	Ch	SU
*Abutilonpannosum* (G. Forst.) Schltdl.	Malvaceae	1	1	Ch	TR
*Abutilonramiflorum* A.St.-Hil.	Malvaceae	1	0	Th	TR
*Achnatherumparviflorum* (Desf.) M.Nobis	Poaceae	1	1	He	IT+SA
*Aeluropuslagopoides* (L.) Thwaites	Poaceae	0	1	Ch	IT+SA
*Aervajavanica* Juss.	Amaranthaceae	1	1	Th	TR
*Aervalanata* (L.) Juss.	Amaranthaceae	0	1	Th	TR
*Aizooncanariense* L.	Aizoaceae	1	1	Th	SU
*Amaranthusalbus* L.	Amaranthaceae	1	1	Th	AM
*Anabasissetifera* Moq.	Chenopodiacea	0	1	Ch	SA
*Anastaticahierochuntica* L.	Brassicaceae	1	1	Th	SA
*Anchusamilleri* Lam. ex Spreng.	Boraginaceae	1	0	Th	SA
*Andrachneaspera* Spreng.	Euphorbiaceae	1	0	Ch	SU
*Arnebiahispidissima* (Lehm.) DC.	Boraginaceae	0	1	Th	SA+SU
*Asphodelustenuifolius* Cav.	Asphodelaceae	1	1	Ge	SA+SU
*Astragalusannularis* Forssk.	Fabaceae	1	1	Th	SA
*Astragaluscrenatus* Schult.	Fabaceae	1	1	Th	SA
*Astragalustribuloides* Delile	Fabaceae	1	1	Th	SA+IT
*Astragalusvogelii* (Webb) Bornm.	Fabaceae	1	1	Th	SA
*Boerhaviaboissieri* Heimerl	Nyctaginaceae	1	1	Th	SU
*Boerhaviadiffusa* L.	Nyctaginaceae	1	1	Ch	SA+TR
*Boerhaviarepens* L.	Nyctaginaceae	1	1	Ch	TR
*Brachiariaeruciformis* (Sm.) Griseb.	Poaceae	1	1	Th	TR
*Cadabafarinosa* Frossk.	Capparaceae	1	1	Ch	SU
*Cadabaglandulosa* Frossk.	Capparaceae	1	0	Ph	TR
*Calotropisprocera* (Aiton) Dryand.	Apocynaceae	1	1	Ph	SA
*Capparisdecidua* Edgew.	Capparaceae	1	1	Ph	SA+SU
*Capparisspinosa* L.	Capparaceae	1	1	Ch	ME
*Cenchrusciliaris* L.	Poaceae	1	1	He	SA+SU
*Cenchrusdivisus* (J.F.Gmel.) Verloove, Govaerts & Buttler	Poaceae	1	1	He	SA
*Cenchruspennisetiformis* Steud.	Poaceae	1	1	Th	AM
*Cenchrussetaceus* (Forssk.) Morrone	Poaceae	1	1	He	SA
*Chenopodiummurale* L.	Chenopodiacea	1	0	Th	Cosm
*Cissusquadrangularis* L.	Vitaceae	1	0	Ch	SA+SU
*Citrulluscolocynthis* (L.) Schrad.	Cucurbitaceae	1	1	Th	SA
*Cleomeamblyocarpa* Barratte & Murb.	Cleomaceae	1	0	Ch	SA+SU
*Cleomebrachycarpa* Vahl ex DC.	Cleomaceae	1	0	Ch	SA
*Cleomedroserifolia* (Forssk.) Delile	Cleomaceae	1	0	Ch	SU
*Cleomepallida* Kotschy	Cleomaceae	1	1	Th	SU
*Cleomeparadoxa* R. Br. ex DC.	Cleomaceae	1	0	Ch	SU
*Cocculuspendulus* (J.R. & G. Forst.) Diels	Menispermaceae	1	0	Ch	SU
*Cometessurattensis* Burm.f.	Caryophyllaceae	1	0	Th	SU
*Commiphoragileadensis* (L.) C.Chr.	Burseraceae	1	0	Ph	SU
*Commiphoraquadricincta* Schweinf	Burseraceae	1	0	Ph	Su
*Convolvulusdeserti* Hochst. & Steud.	Convolvulaceae	1	1	Ch	SA
*Corbichoniadecumbens* (Forssk.) Exell	Lophiocarpaceae	1	0	He	SA+ SU
*Corchorusdepressus* (L.) Stocks	Tiliaceae	1	1	Ch	ME+SA
*Crotalariamicrophylla* Vahl	Fabaceae	1	1	Th	SA+SU
*Cymbopogonschoenanthus* (L.) Spreng.	Poaceae	1	0	Th	SA
*Cynanchumboveanum* Decne.	Apocynaceae	1	0	He	SU
*Cynanchumradians* (Forssk.) Lam.	Apocynaceae	0	1	Ch	TR
*Cynodondactylon* (L.) Pers.	Poaceae	1	1	Ge	Cosm
*Cyperusconglomeratus* Rottb.	Cyperaceae	1	1	Ge	SA
*Cyperuslaevigatus* L.	Cyperaceae	1	1	Ge	Cosm
*Dactylocteniumaegyptium* (L.) Willd.	Poaceae	0	1	Th	Cosm
*Daturainnoxia* Mill.	Solanaceae	0	1	Th	Cosm
*Desmidorchisretrospiciens* Ehrenb.	Apocynaceae	1	0	Th	SA+SU
*Dicomatomentosa* Cass.	Asteraceae	1	0	Ch	SU
*Echinopshussonii* Boiss.	Asteraceae	1	0	He	SA+ME
*Ephedraalata* Decne.	Ephedraceae	1	0	Ch	SA+I-T
*Erythrococcaabyssinica* Pax.	Euphorbiaceae	1	1	Th	TR
*Euphorbiaarabica* Hochst. & Steud. ex T. Anderson	Euphorbiaceae	1	1	Th	SA
*Euphorbiacuneata* Vahl	Euphorbiaceae	1	0	Ph	SA+SU
*Euphorbiagranulata* Frossk.	Euphorbiaceae	1	1	Th	SU
*Euphorbiahirta* L.	Euphorbiaceae	1	1	Th	Cosm
*Euphorbiainaequilatera* Sond.	Euphorbiaceae	1	1	Th	SU
*Euphorbiaprostrata* Aiton	Euphorbiaceae	1	1	Th	Cosm
*Euphorbiaserpens* Kunth	Euphorbiaceae	1	1	Th	AM
*Farsetialongisiliqua* Decne	Brassicaceae	1	1	Ch	SU
*Farsetiastylosa* R. Br.	Brassicaceae	1	1	Ch	SU
*Forsskaoleatenacissima* L.	Urticaceae	1	1	Ch	SA+SU
*Gisekiapharnaceoides* L.	Aizoaceae	1	1	Th	TR
*Grewiaerythraea* Schweinf.	Malvaceae	1	0	Ph	SA+ SU
*Gypsophilacapillaris* C.Chr.	Caryophyllaceae	1	0	Ch	IT
*Haloxylonsalicornicum* (Mo.) Bun. ex Boiss.	Chenopodiacea	0	1	Ch	IT
*Heliotropiumarbainense* Fresen.	Boraginaceae	1	1	Ch	SA
*Heliotropiumbacciferum* Forssk.	Boraginaceae	1	1	Ch	SA+SU
*Heliotropiumdigynum* Asch. ex C.Chr.	Boraginaceae	1	1	Ch	SA
*Indigoferaschimperi* Jaub. & Spach	Fabaceae	1	0	Th	SA+IT
*Indigoferaspinosa* Frossk	Fabaceae	1	1	Th	ME
*Kickxiafloribunda* (Boiss.) Täckh. & Boulos	Scrophulariaceae	1	1	Ch	SA
*Lasiurusscindicus* Henrard	Poaceae	1	1	He	SU
*Launaeacapitata* (Spreng.) Dandy	Asteraceae	1	1	Th	SA
*Lavandulacoronopifolia* Poir.	Lamiaceae	1	0	Ch	SA+ SU
*Leptadeniapyrotechnica* (Forssk.) Decne.	Apocynaceae	1	1	Ph	SA+SU
*Lindenbergiaindica* (L.) Vatke	Scrophulariaceae	1	0	Th	SA
*Linumbienne* Mill.	Linaceae	1	0	Ch	SU
*Lyciumshawii* Roem. & Schult.	Solanaceae	1	0	He	SA+SU
*Maeruacrassifolia* Frossk.	Capparaceae	1	0	Ph	SA+SU
*Maeruaoblongifolia* A.Rich.	Capparaceae	1	1	Ph	SU
*Malvaparviflora* L.	Malvaceae	1	1	Ch	ME+IT
*Monsoniaheliotropioides* (Cav.) Boiss.	Geraniaceae	1	0	Th	SU
*Morettiacanescens* Boiss.	Brassicaceae	1	1	Ch	ME
*Moringaperegrina* (Forssk.) Fiori	Moringaceae	1	0	Ph	SU
*Neltumajuliflora* (Sw.) Raf.	Fabaceae	0	1	Ph	SA
*Ochradenusarabicus* Chaudhary, Hillc. & A.G.Mill	Resedaceae	1	1	Th	SA
*Ochradenusbaccatus* Del.	Resedaceae	1	1	Th	SA
*Oligomerislinifolia* (Fahl ex Hornen) J.F. Macbr	Resedaceae	1	0	Th	ME+IT
*Onobrychisptolemaica* (Delile) DC.	Fabaceae	1	1	Ch	SU
*Otostegiafruticosa* Schweinf. ex Penz.	Lamiaceae	1	0	Th	SA+ SU
*Oxystelmaesculentum* (L.f.) Sm.	Apocynaceae	1	1	He	TR
*Panicumturgidum* Forssk.	Poaceae	1	1	He	SA+SU
*Pergulariatomentosa* L.	Apocynaceae	1	1	Ch	SA+SU
*Phyllanthusrotundifolius* J.G.Klein ex Willd.	Phyllanthaceae	1	1	Th	Cosm
*Polygalaerioptera* DC.	Polygalaceae	1	0	Th	Sa
*Polygonumequisetiforme* Sm.	Polygalaceae	0	1	He	ME+IT
*Portulacaoleracea* L.	Portulacaeae	1	1	Th	Cosm
*Pulicariaorientalis* Jaub. & Spach	Asteraceae	1	0	Ch	SA+SU
*Resedaluteola* L.	Resedaceae	1	0	Th	ME+IT
*Rhazyastricta* Decne.	Apocynaceae	0	1	Ch	SA+SU
*Rumexvesicarius* L.	Polygonaceae	1	1	Th	SA
*Salsolaimbricata* Forssk.	Chenopodiacea	1	1	Ph	SU
*Salsolatetrandra* Forssk.	Chenopodiacea	1	1	Ch	SA
*Salviaaegyptiaca* L.	Lamiaceae	1	0	Ch	SA+ SU
*Salviadeserti* Decne.	Lamiaceae	1	0	Ch	SA+ SU
*Schouwiapurpurea* (F0rssk.) Schweinf.	Brassicaceae	0	1	Th	ME+SA
*Schweinfurthiapterosperma* (A.Rich.) A.Braun	Plantaginaceae	1	0	Ch	SU+SA
*Scorzoneratortuosissima* Boiss	Asteraceae			Ch	SA+SU
*Scrophulariaarguta* Aiton	Scrophulariaceae	1	0	Th	SU+SA
*Senegaliahamulosa* (Benth.) Boatwr.	Fabaceae	1	0	Ph	SU
*Sennaitalica* Mill.	Fabaceae	1	1	Ch	SU
*Setariaverticillata* (L.) P. Beauv.	Poaceae	1	1	Th	Cosm
*Solanumincanum* L.	Solanaceae	1	1	Th	SU
*Sorghumhalepense* (L.) Pers.	Poaceae	1	0	Th	TR
*Stipadregeana* Steud.	Poaceae	1	1	Th	IT+SA
*Stipagrostisplumosa* (L.) Mun. ex T. And.	Poaceae	1	1	He	IT+SA
*Suaedavermiculata* Forssk. ex J.F.Gmel.	Chenopodiacea	0	1	Ch	SA
*Tephrosianubica* (Boiss.) Baker	Fabaceae	1	1	Th	SA
Tephrosiapurpureasubsp.apollinea (Delile) Hosni & El-Karemy	Fabaceae	1	1	Ch	SU
*Trianthemacrystallina* Vahl	Aizoaceae	1	0	Th	TR
*Tribulopispentandra* R.Br.	Zygophyllaceae	1	1	Ch	SA
*Tribulusmacropterus* Boiss.	Zygophyllaceae	1	1	Th	SU
*Tribuluspentandrus* Forssk.	Zygophyllaceae	0	1	Ch	SA
*Tribuluspterocarpus* Ehrenb. ex Müll. Berol	Zygophyllaceae	0	1	Ch	SU
*Tribulusterrestris* L.	Zygophyllaceae	1	1	Th	ME+SU
*Trichodesmaafricanum* (L.) Sm.	Boraginaceae	1	0	Th	SA
*Vachelliaflava* (Forssk.) Kyal. & Boatwr.	Fabaceae	0	1	Ph	SU
*Vachelliatortilis* (Forssk.) Galasso & Banfi	Fabaceae	0	1	Ph	SU
*Zaleyapentandra* (L.) C. Jeffrey	Aizoaceae	1	1	Th	SU
*Ziziphusspinachristi* (L.) Desf	Rhamnaceae	0	1	Ph	SA+SU
*Zygophyllumcoccineum* L.	Zygophyllaceae	0	1	Ch	SA
*Zygophyllumindicum* (Burm.f.) Christenh. & Byng	Zygophyllaceae	1	1	Ch	SA
*Zygophyllummolle* (Delile) Christenh. & Byng	Zygophyllaceae	1	1	Ch	SA
*Zygophyllumsimplex* L.	Zygophyllaceae	1	1	Ch	SA
